# {*N*′-[1-(2-Pyrid­yl)ethyl­idene-κ*N*]benzo­hydrazidato-κ^2^ *N*′,*O*}{*N*′-[1-(2-pyrid­yl)ethyl­idene-κ*N*]benzohydrazide-κ^2^ *N*′,*O*}copper(II) trichloro­acetate

**DOI:** 10.1107/S1600536811036592

**Published:** 2011-09-14

**Authors:** Amitabha Datta, Nien-Tsu Chuang, Yun-Sheng Wen, Jui-Hsien Huang, Shiann-Cherng Sheu

**Affiliations:** a Department of Chemistry, National Changhua University of Education, Changhua, 50058, Taiwan; b Institute of Chemistry, Academia Sinica, Nankang, Taipei 115, Taiwan; c Department of Occupational Health and Safety, Chang Jung Christian University, Tainan City, 71101, Taiwan

## Abstract

In the title complex, [Cu(C_14_H_13_N_3_O)(C_14_H_12_N_3_O)](CCl_3_COO), the central Cu(II) ion exhibits a distorted octa­hedral geometry with the two ligands coordinating in an meridional format. The N_4_O_2_ ligand environment is defined by two benzoyl O atoms, two pyridyl N atoms and imino N atoms. As evidenced by the bond lengths, the two benzohydrazone ligands exist in distinctively different forms, one of them as a regular neutral ligand and the other as an anionic enolate arising from deprotonation. The much longer Cu—O bond and longer Cu—N bond lengths in the neutral benzohydrazone ligand imply weak ligation in comparison with the anionic enolate form. The acute angles of the five-membered rings cause a significant deviation from a regular octa­hedral geometry.

## Related literature

For related complexes of the same precursor ligand, see: Patole *et al.* (2003 ▶); Sen *et al.* (2005 ▶, 2007*a* ▶,*b* ▶); Ray *et al.* (2008 ▶); Datta *et al.* (2010 ▶). 
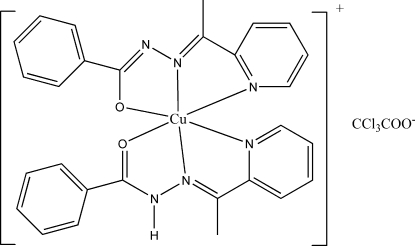


## Experimental

### 

#### Crystal data

[Cu(C_14_H_13_N_3_O)(C_14_H_12_N_3_O)](C_2_Cl_3_O_2_)
*M* *_r_* = 703.45
Triclinic, 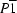

*a* = 8.3341 (6) Å
*b* = 13.0470 (9) Å
*c* = 16.0729 (12) Å
α = 107.737 (1)°
β = 102.541 (1)°
γ = 95.259 (1)°
*V* = 1601.3 (2) Å^3^
*Z* = 2
Mo *K*α radiation
μ = 0.98 mm^−1^
*T* = 295 K
0.30 × 0.27 × 0.25 mm

#### Data collection

Bruker APEXII CCD area-detector diffractometer
Absorption correction: multi-scan (*SADABS*; Sheldrick, 1996 ▶) *T*_min_ = 0.799, *T*_max_ = 0.875
9034 measured reflections
6180 independent reflections
4962 reflections with *I* > 2σ(*I*)
*R*_int_ = 0.016

#### Refinement

*R*[*F* ^2^ > 2σ(*F* ^2^)] = 0.059
*wR*(*F* ^2^) = 0.189
*S* = 1.03
6180 reflections
399 parameters
1 restraint
H-atom parameters constrained
Δρ_max_ = 1.16 e Å^−3^
Δρ_min_ = −0.52 e Å^−3^

### 

Data collection: *APEX2* (Bruker, 2007 ▶); cell refinement: *SAINT* (Bruker, 2007 ▶); data reduction: *SAINT*; program(s) used to solve structure: *SHELXS97* (Sheldrick, 2008 ▶); program(s) used to refine structure: *SHELXL97* (Sheldrick, 2008 ▶); molecular graphics: *SHELXTL* (Sheldrick, 2008 ▶); software used to prepare material for publication: *SHELXTL*.

## Supplementary Material

Crystal structure: contains datablock(s) I, global. DOI: 10.1107/S1600536811036592/zk2027sup1.cif

Structure factors: contains datablock(s) I. DOI: 10.1107/S1600536811036592/zk2027Isup2.hkl

Additional supplementary materials:  crystallographic information; 3D view; checkCIF report

## Figures and Tables

**Table 1:** Selected bond lengths (Å)

Cu—N1	2.046 (3)
Cu—N2	1.938 (3)
Cu—N4	2.196 (3)
Cu—N5	2.088 (3)
Cu—O1	1.996 (3)
Cu—O2	2.420 (2)

**Fig. 1.**
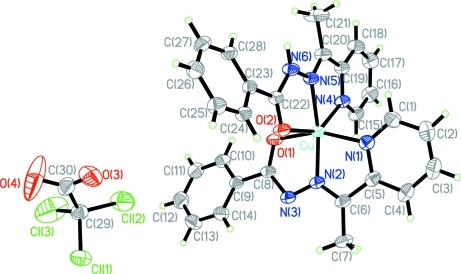
The molecular structure of the title complex, showing 30% displacement ellipsoids.

## supplementary crystallographic information

### Comment

The title complex is consisted of two tridentate benzohydrazone ligands oriented in a meridional fashion. One ligand coordinates in the deprotonated enolate form and acts as a monoanion. The other ligand coordinates in the regular neutral form. The nonequivalent coordination of these two ligands is proved by the bond distances surrounding the cuprous ion. The Cu—O distance for neutral ligand (2.420 (2) Å) is much longer than that of deprotonated ligand, 1.996 (3) Å. Contrarily, the C—O bond distance of the neutral ligand (1.228 (4) Å) is shorter than that of the deprotonated enolate form (1.280 (4) Å). The Cu—N bond distances of the enolate ligand (2.046 (3) and 1.938 (3) Å) are also shorter than those of the neutral ligand (2.196 (3) and 2.088 (3) Å). Nevertheless, the Cu—O and Cu—N bond distances (Table 1) are comparable with the literature reported complexes under the same ligand mode(Patole *et al.*, 2003, Sen *et al.*, 2005, 2007*a*,b, Ray *et al.*, 2008. Datta *et al.*, 2010). The distortion from regular octahedral symmetry is relatively large considering that the bond angles surrounding cuprous ion lie between 71.3 (1) and 163.0 (1)°. The equatorial plane can be defined by O1, N1, N2 and N5 atoms and, accordingly, the axial sites are occupied by O2 and N4 atoms. The Cu(II) ion deviates from the equatorial plane towards the axial N4 atom by 0.1421 (4) Å. The dihedral angles between two pyridine rings and two benzene rings are 86.1 (2)° and 81.7 (2)°, respectively.

### Experimental

The ligand precursor, [C_6_H_5_C(O)NHN=C(CH_3_)C_5_H_4_N] was prepared according to a literature procedure (Sen *et al.*, 2005). To the ligand (2 mmol), methanolic solution (20 ml) of anhydrous copper trichloroacetate (0.388 g, 1 mmol) was added with constant stirring and was kept at room temperature yielding light green square-shaped crystals suitable for X-ray diffraction after few days. Crystals were filtered and were air-dried.

### Refinement

All the H atoms were positioned geometrically and refined as riding atoms, with C_aryl_—H = 0.93, C_methyl_—H = 0.96 Å, while *Uiso*(H) = 1.5 *Ueq*(C) for the methyl H atoms and 1.2 *Ueq* (C) for all the other H atoms are used in the final refinement. A disagreeable reflection with delta(F2)/ e.s.d. >10 was omitted.

### Crystal data

**Table:**

[Cu(C_14_H_13_N_3_O)(C_14_H_12_N_3_O)](C_2_Cl_3_O_2_)	*V* = 1601.3 (2) Å^3^
*M**_r_* = 703.45	*Z* = 2
Triclinic, *P*1	*F*(000) = 718
Hall symbol: -P 1	*D*_x_ = 1.459 Mg m^−^^3^
*a* = 8.3341 (6) Å	Mo *K*α radiation, λ = 0.71073 Å
*b* = 13.0470 (9) Å	µ = 0.98 mm^−^^1^
*c* = 16.0729 (12) Å	*T* = 295 K
α = 107.737 (1)°	Square, green
β = 102.541 (1)°	0.30 × 0.27 × 0.25 mm
γ = 95.259 (1)°	

### Data collection

**Table:**

Bruker APEXII CCD area-detector diffractometer	6180 independent reflections
Radiation source: fine-focus sealed tube	4962 reflections with *I* > 2σ(*I*)
graphite	*R*_int_ = 0.016
phi and ω scans	θ_max_ = 26.0°, θ_min_ = 2.5°
Absorption correction: multi-scan (*SADABS*; Sheldrick, 1996)	*h* = −10→10
*T*_min_ = 0.799, *T*_max_ = 0.875	*k* = −15→16
9034 measured reflections	*l* = −17→19

### Refinement

**Table:**

Refinement on *F*^2^	Primary atom site location: structure-invariant direct methods
Least-squares matrix: full	Secondary atom site location: difference Fourier map
*R*[*F*^2^ > 2σ(*F*^2^)] = 0.059	Hydrogen site location: inferred from neighbouring sites
*wR*(*F*^2^) = 0.189	H-atom parameters constrained
*S* = 1.03	*w* = 1/[σ^2^(*F*_o_^2^) + (0.1266*P*)^2^ + 0.7166*P*] where *P* = (*F*_o_^2^ + 2*F*_c_^2^)/3
6180 reflections	(Δ/σ)_max_ = 0.001
399 parameters	Δρ_max_ = 1.16 e Å^−^^3^
1 restraint	Δρ_min_ = −0.52 e Å^−^^3^

### Special details

**Table:**

Geometry. All e.s.d.'s (except the e.s.d. in the dihedral angle between two l.s. planes) are estimated using the full covariance matrix. The cell e.s.d.'s are taken into account individually in the estimation of e.s.d.'s in distances, angles and torsion angles; correlations between e.s.d.'s in cell parameters are only used when they are defined by crystal symmetry. An approximate (isotropic) treatment of cell e.s.d.'s is used for estimating e.s.d.'s involving l.s. planes.
Refinement. Refinement of *F*^2^ against ALL reflections. The weighted *R*-factor *wR* and goodness of fit *S* are based on *F*^2^, conventional *R*-factors *R* are based on *F*, with *F* set to zero for negative *F*^2^. The threshold expression of *F*^2^ > σ(*F*^2^) is used only for calculating *R*-factors(gt) *etc*. and is not relevant to the choice of reflections for refinement. *R*-factors based on *F*^2^ are statistically about twice as large as those based on *F*, and *R*- factors based on ALL data will be even larger.

### Fractional atomic coordinates and isotropic or equivalent isotropic displacement parameters (Å^2^)

**Table:**

	*x*	*y*	*z*	*U*_iso_*/*U*_eq_	
C1	0.8662 (5)	0.7231 (4)	0.8172 (3)	0.0642 (10)	
H1	0.9207	0.7952	0.8428	0.077*	
C2	0.8171 (6)	0.6703 (5)	0.8729 (3)	0.0779 (12)	
H2	0.8365	0.7069	0.9346	0.094*	
C3	0.7401 (6)	0.5639 (5)	0.8359 (4)	0.0810 (14)	
H3	0.7067	0.5271	0.8722	0.097*	
C4	0.7121 (5)	0.5114 (4)	0.7440 (3)	0.0707 (12)	
H4	0.6604	0.4387	0.7180	0.085*	
C5	0.7614 (4)	0.5676 (3)	0.6908 (3)	0.0524 (8)	
C6	0.7387 (4)	0.5210 (3)	0.5918 (3)	0.0521 (8)	
C7	0.6495 (6)	0.4074 (3)	0.5377 (3)	0.0740 (12)	
H7A	0.6098	0.4019	0.4755	0.111*	
H7B	0.5568	0.3907	0.5606	0.111*	
H7C	0.7247	0.3567	0.5422	0.111*	
C8	0.8707 (4)	0.6479 (3)	0.4554 (2)	0.0478 (7)	
C9	0.8752 (4)	0.6359 (3)	0.3610 (2)	0.0501 (8)	
C10	1.0003 (5)	0.7006 (3)	0.3449 (3)	0.0596 (9)	
H10	1.0787	0.7523	0.3933	0.072*	
C11	1.0075 (6)	0.6879 (4)	0.2581 (3)	0.0723 (11)	
H11	1.0934	0.7292	0.2479	0.087*	
C12	0.8891 (7)	0.6145 (4)	0.1860 (3)	0.0810 (13)	
H12	0.8925	0.6080	0.1272	0.097*	
C13	0.7658 (7)	0.5509 (4)	0.2012 (3)	0.0834 (14)	
H13	0.6871	0.5002	0.1524	0.100*	
C14	0.7575 (5)	0.5614 (3)	0.2868 (3)	0.0642 (10)	
H14	0.6723	0.5183	0.2960	0.077*	
C15	1.2778 (5)	0.7146 (3)	0.7063 (3)	0.0568 (9)	
H15	1.2450	0.6421	0.6691	0.068*	
C16	1.4413 (5)	0.7497 (4)	0.7564 (3)	0.0677 (11)	
H16	1.5173	0.7016	0.7527	0.081*	
C17	1.4910 (5)	0.8573 (4)	0.8123 (3)	0.0693 (11)	
H17	1.6000	0.8826	0.8478	0.083*	
C18	1.3744 (5)	0.9265 (3)	0.8142 (3)	0.0622 (10)	
H18	1.4044	0.9996	0.8505	0.075*	
C19	1.2123 (4)	0.8857 (3)	0.7613 (2)	0.0490 (8)	
C20	1.0801 (4)	0.9549 (3)	0.7556 (3)	0.0533 (8)	
C21	1.1254 (6)	1.0762 (3)	0.8015 (4)	0.0950 (19)	
H21A	1.2375	1.1002	0.8011	0.142*	
H21B	1.1173	1.0944	0.8628	0.142*	
H21C	1.0503	1.1118	0.7700	0.142*	
C22	0.6592 (4)	0.9005 (3)	0.6336 (2)	0.0445 (7)	
C23	0.5230 (4)	0.9641 (3)	0.6162 (2)	0.0428 (7)	
C24	0.3589 (4)	0.9092 (3)	0.5859 (2)	0.0496 (8)	
H24	0.3367	0.8353	0.5785	0.060*	
C25	0.2288 (5)	0.9639 (3)	0.5669 (3)	0.0595 (9)	
H25	0.1193	0.9275	0.5484	0.071*	
C26	0.2622 (5)	1.0744 (3)	0.5757 (3)	0.0634 (10)	
H26	0.1751	1.1113	0.5622	0.076*	
C27	0.4247 (5)	1.1279 (3)	0.6045 (3)	0.0624 (10)	
H27	0.4472	1.2013	0.6105	0.075*	
C28	0.5548 (5)	1.0737 (3)	0.6245 (3)	0.0520 (8)	
H28	0.6641	1.1107	0.6437	0.062*	
C29	0.3176 (6)	0.7394 (4)	0.0606 (3)	0.0661 (10)	
C30	0.2374 (6)	0.8395 (4)	0.1034 (4)	0.0753 (12)	
N1	0.8380 (4)	0.6743 (3)	0.7289 (2)	0.0526 (7)	
N2	0.8012 (3)	0.5880 (2)	0.5580 (2)	0.0468 (6)	
N3	0.7924 (4)	0.5618 (2)	0.4679 (2)	0.0521 (7)	
N4	1.1651 (3)	0.7807 (2)	0.7093 (2)	0.0478 (6)	
N5	0.9358 (3)	0.9034 (2)	0.70525 (19)	0.0456 (6)	
N6	0.8058 (3)	0.9593 (2)	0.6930 (2)	0.0500 (7)	
H6	0.8162	1.0281	0.7214	0.060*	
O1	0.9407 (3)	0.73837 (19)	0.51784 (18)	0.0564 (6)	
O2	0.6418 (3)	0.80149 (19)	0.59581 (19)	0.0558 (6)	
O3	0.2322 (5)	0.8536 (3)	0.1794 (3)	0.1018 (12)	
O4	0.1930 (14)	0.8931 (8)	0.0589 (6)	0.261 (5)	
Cu	0.90049 (5)	0.73603 (3)	0.63528 (3)	0.05047 (18)	
Cl1	0.16329 (19)	0.61979 (11)	0.01824 (10)	0.0956 (4)	
Cl2	0.4774 (2)	0.71845 (14)	0.14101 (15)	0.1265 (7)	
Cl3	0.3983 (5)	0.7551 (2)	−0.02666 (19)	0.1990 (15)	

### Atomic displacement parameters (Å^2^)

**Table:**

	*U*^11^	*U*^22^	*U*^33^	*U*^12^	*U*^13^	*U*^23^
C1	0.057 (2)	0.068 (2)	0.061 (2)	0.0066 (18)	0.0097 (18)	0.017 (2)
C2	0.078 (3)	0.097 (4)	0.063 (3)	0.021 (3)	0.013 (2)	0.034 (3)
C3	0.081 (3)	0.096 (4)	0.087 (3)	0.021 (3)	0.023 (3)	0.059 (3)
C4	0.071 (3)	0.067 (3)	0.091 (3)	0.017 (2)	0.024 (2)	0.047 (2)
C5	0.0419 (17)	0.0469 (18)	0.074 (2)	0.0123 (14)	0.0144 (16)	0.0269 (17)
C6	0.0478 (18)	0.0364 (17)	0.070 (2)	0.0089 (14)	0.0104 (16)	0.0179 (16)
C7	0.085 (3)	0.0380 (19)	0.088 (3)	−0.0057 (19)	0.010 (2)	0.018 (2)
C8	0.0435 (17)	0.0360 (16)	0.059 (2)	0.0079 (13)	0.0117 (15)	0.0091 (14)
C9	0.0549 (19)	0.0355 (16)	0.057 (2)	0.0135 (14)	0.0140 (16)	0.0098 (14)
C10	0.064 (2)	0.0476 (19)	0.066 (2)	0.0114 (17)	0.0211 (19)	0.0144 (17)
C11	0.087 (3)	0.065 (3)	0.074 (3)	0.018 (2)	0.036 (2)	0.024 (2)
C12	0.110 (4)	0.077 (3)	0.064 (3)	0.024 (3)	0.034 (3)	0.025 (2)
C13	0.103 (4)	0.069 (3)	0.057 (3)	0.011 (3)	0.005 (2)	0.003 (2)
C14	0.066 (2)	0.052 (2)	0.064 (2)	0.0067 (18)	0.0079 (19)	0.0127 (18)
C15	0.056 (2)	0.0482 (19)	0.071 (2)	0.0183 (16)	0.0217 (18)	0.0201 (18)
C16	0.054 (2)	0.068 (3)	0.088 (3)	0.0254 (19)	0.021 (2)	0.031 (2)
C17	0.044 (2)	0.077 (3)	0.082 (3)	0.0107 (19)	0.0065 (19)	0.026 (2)
C18	0.0436 (19)	0.060 (2)	0.072 (2)	0.0040 (16)	0.0085 (17)	0.0109 (19)
C19	0.0405 (17)	0.0453 (18)	0.058 (2)	0.0058 (13)	0.0128 (14)	0.0125 (15)
C20	0.0430 (18)	0.0400 (17)	0.065 (2)	0.0068 (14)	0.0089 (16)	0.0049 (16)
C21	0.056 (2)	0.045 (2)	0.143 (5)	0.0061 (18)	0.001 (3)	−0.008 (3)
C22	0.0430 (16)	0.0393 (16)	0.0497 (18)	0.0053 (13)	0.0122 (14)	0.0132 (14)
C23	0.0410 (16)	0.0403 (16)	0.0454 (17)	0.0072 (12)	0.0109 (13)	0.0121 (13)
C24	0.0452 (17)	0.0432 (17)	0.058 (2)	0.0037 (14)	0.0114 (15)	0.0161 (15)
C25	0.0426 (18)	0.060 (2)	0.072 (2)	0.0059 (16)	0.0120 (17)	0.0183 (19)
C26	0.058 (2)	0.062 (2)	0.069 (2)	0.0254 (18)	0.0090 (19)	0.020 (2)
C27	0.069 (2)	0.0408 (18)	0.074 (3)	0.0121 (17)	0.008 (2)	0.0217 (18)
C28	0.0500 (19)	0.0409 (17)	0.058 (2)	0.0009 (14)	0.0075 (15)	0.0135 (15)
C29	0.077 (3)	0.059 (2)	0.062 (2)	0.008 (2)	0.025 (2)	0.0147 (19)
C30	0.086 (3)	0.066 (3)	0.082 (3)	0.024 (2)	0.022 (3)	0.030 (2)
N1	0.0419 (15)	0.0540 (17)	0.0612 (18)	0.0060 (12)	0.0107 (13)	0.0206 (14)
N2	0.0452 (15)	0.0338 (13)	0.0583 (17)	0.0058 (11)	0.0126 (12)	0.0118 (12)
N3	0.0571 (17)	0.0368 (14)	0.0568 (17)	0.0048 (12)	0.0127 (14)	0.0099 (12)
N4	0.0423 (14)	0.0393 (14)	0.0611 (17)	0.0074 (11)	0.0156 (13)	0.0143 (13)
N5	0.0413 (14)	0.0385 (14)	0.0512 (15)	0.0087 (11)	0.0106 (12)	0.0073 (12)
N6	0.0418 (14)	0.0362 (14)	0.0612 (17)	0.0111 (11)	0.0069 (12)	0.0043 (12)
O1	0.0632 (15)	0.0387 (12)	0.0574 (15)	−0.0042 (11)	0.0175 (12)	0.0046 (11)
O2	0.0510 (13)	0.0346 (12)	0.0705 (16)	0.0058 (10)	0.0059 (12)	0.0087 (11)
O3	0.109 (3)	0.096 (3)	0.079 (2)	0.042 (2)	0.023 (2)	−0.008 (2)
O4	0.395 (13)	0.307 (10)	0.265 (9)	0.269 (10)	0.187 (9)	0.224 (9)
Cu	0.0526 (3)	0.0376 (3)	0.0532 (3)	−0.00343 (18)	0.0129 (2)	0.00748 (19)
Cl1	0.0939 (9)	0.0706 (8)	0.0948 (9)	−0.0064 (6)	0.0015 (7)	0.0103 (7)
Cl2	0.0788 (9)	0.0975 (11)	0.1661 (17)	0.0305 (8)	−0.0130 (10)	0.0181 (11)
Cl3	0.336 (4)	0.1402 (18)	0.171 (2)	0.017 (2)	0.189 (3)	0.0433 (16)

### Geometric parameters (Å, °)

**Table:**

Cu—N1	2.046 (3)	C14—H14	0.9300
N2—N3	1.367 (4)	C15—N4	1.330 (4)
Cu—N2	1.938 (3)	C15—C16	1.378 (6)
Cu—N4	2.196 (3)	C15—H15	0.9300
N5—N6	1.373 (4)	C16—C17	1.383 (6)
Cu—N5	2.088 (3)	C16—H16	0.9300
N6—H6	0.8600	C17—C18	1.385 (6)
Cu—O1	1.996 (3)	C17—H17	0.9300
Cu—O2	2.420 (2)	C18—C19	1.388 (5)
C1—N1	1.324 (5)	C18—H18	0.9300
C1—C2	1.389 (6)	C19—N4	1.338 (4)
C1—H1	0.9300	C19—C20	1.493 (5)
C2—C3	1.364 (7)	C20—N5	1.285 (4)
C2—H2	0.9300	C20—C21	1.500 (5)
C3—C4	1.383 (7)	C21—H21A	0.9600
C3—H3	0.9300	C21—H21B	0.9600
C4—C5	1.385 (6)	C21—H21C	0.9600
C4—H4	0.9300	C22—O2	1.228 (4)
C5—N1	1.367 (5)	C22—N6	1.367 (4)
C5—C6	1.481 (6)	C22—C23	1.494 (4)
C6—N2	1.289 (5)	C23—C28	1.390 (5)
C6—C7	1.496 (5)	C23—C24	1.393 (5)
C7—H7A	0.9600	C24—C25	1.382 (5)
C7—H7B	0.9600	C24—H24	0.9300
C7—H7C	0.9600	C25—C26	1.400 (6)
O1—C8	1.280 (4)	C25—H25	0.9300
C8—N3	1.338 (4)	C26—C27	1.376 (6)
C8—C9	1.485 (5)	C26—H26	0.9300
C9—C14	1.393 (5)	C27—C28	1.381 (5)
C9—C10	1.398 (5)	C27—H27	0.9300
C10—C11	1.370 (6)	C28—H28	0.9300
C10—H10	0.9300	C29—C30	1.561 (6)
C11—C12	1.374 (7)	C29—Cl3	1.738 (4)
C11—H11	0.9300	C29—Cl2	1.747 (5)
C12—C13	1.370 (7)	C29—Cl1	1.777 (5)
C12—H12	0.9300	C30—O4	1.173 (7)
C13—C14	1.360 (7)	C30—O3	1.189 (6)
C13—H13	0.9300		
			
N1—C1—C2	122.2 (4)	C20—C21—H21C	109.5
N1—C1—H1	118.9	H21A—C21—H21C	109.5
C2—C1—H1	118.9	H21B—C21—H21C	109.5
C3—C2—C1	119.1 (5)	O2—C22—N6	121.8 (3)
C3—C2—H2	120.5	O2—C22—C23	122.1 (3)
C1—C2—H2	120.5	N6—C22—C23	116.1 (3)
C2—C3—C4	119.4 (4)	C28—C23—C24	119.3 (3)
C2—C3—H3	120.3	C28—C23—C22	122.4 (3)
C4—C3—H3	120.3	C24—C23—C22	118.2 (3)
C3—C4—C5	119.6 (4)	C25—C24—C23	120.3 (3)
C3—C4—H4	120.2	C25—C24—H24	119.9
C5—C4—H4	120.2	C23—C24—H24	119.9
N1—C5—C4	120.3 (4)	C24—C25—C26	120.0 (3)
N1—C5—C6	114.8 (3)	C24—C25—H25	120.0
C4—C5—C6	125.0 (4)	C26—C25—H25	120.0
N2—C6—C5	113.4 (3)	C27—C26—C25	119.5 (3)
N2—C6—C7	124.2 (4)	C27—C26—H26	120.3
C5—C6—C7	122.4 (3)	C25—C26—H26	120.3
C6—C7—H7A	109.5	C26—C27—C28	120.7 (3)
C6—C7—H7B	109.5	C26—C27—H27	119.7
H7A—C7—H7B	109.5	C28—C27—H27	119.7
C6—C7—H7C	109.5	C27—C28—C23	120.3 (3)
H7A—C7—H7C	109.5	C27—C28—H28	119.9
H7B—C7—H7C	109.5	C23—C28—H28	119.9
O1—C8—N3	125.2 (3)	C30—C29—Cl3	111.7 (3)
O1—C8—C9	118.4 (3)	C30—C29—Cl2	111.6 (3)
N3—C8—C9	116.4 (3)	Cl3—C29—Cl2	108.5 (3)
C14—C9—C10	118.2 (4)	C30—C29—Cl1	108.9 (3)
C14—C9—C8	121.9 (3)	Cl3—C29—Cl1	109.4 (3)
C10—C9—C8	119.9 (3)	Cl2—C29—Cl1	106.7 (2)
C11—C10—C9	120.1 (4)	O4—C30—O3	127.5 (6)
C11—C10—H10	119.9	O4—C30—C29	117.5 (6)
C9—C10—H10	119.9	O3—C30—C29	115.0 (4)
C10—C11—C12	120.6 (4)	C1—N1—C5	119.5 (3)
C10—C11—H11	119.7	C1—N1—Cu	128.5 (3)
C12—C11—H11	119.7	C5—N1—Cu	112.0 (2)
C13—C12—C11	119.7 (5)	C6—N2—N3	122.9 (3)
C13—C12—H12	120.2	C6—N2—Cu	119.6 (3)
C11—C12—H12	120.2	N3—N2—Cu	117.4 (2)
C14—C13—C12	120.7 (5)	C8—N3—N2	107.9 (3)
C14—C13—H13	119.7	C15—N4—C19	119.1 (3)
C12—C13—H13	119.7	C15—N4—Cu	126.5 (2)
C13—C14—C9	120.7 (4)	C19—N4—Cu	114.4 (2)
C13—C14—H14	119.6	C20—N5—N6	120.0 (3)
C9—C14—H14	119.6	C20—N5—Cu	120.5 (2)
N4—C15—C16	122.4 (4)	N6—N5—Cu	119.4 (2)
N4—C15—H15	118.8	C22—N6—N5	116.8 (3)
C16—C15—H15	118.8	C22—N6—H6	121.6
C15—C16—C17	119.2 (4)	N5—N6—H6	121.6
C15—C16—H16	120.4	C8—O1—Cu	109.8 (2)
C17—C16—H16	120.4	C22—O2—Cu	110.6 (2)
C16—C17—C18	118.4 (4)	N2—Cu—O1	79.25 (11)
C16—C17—H17	120.8	N2—Cu—N1	80.17 (12)
C18—C17—H17	120.8	O1—Cu—N1	159.01 (12)
C17—C18—C19	119.2 (4)	N2—Cu—N5	162.98 (12)
C17—C18—H18	120.4	O1—Cu—N5	100.25 (11)
C19—C18—H18	120.4	N1—Cu—N5	100.64 (12)
N4—C19—C18	121.7 (3)	N2—Cu—N4	122.15 (11)
N4—C19—C20	115.2 (3)	O1—Cu—N4	94.88 (11)
C18—C19—C20	123.1 (3)	N1—Cu—N4	92.52 (11)
N5—C20—C19	115.0 (3)	N5—Cu—N4	74.87 (10)
N5—C20—C21	125.4 (3)	N2—Cu—O2	91.71 (10)
C19—C20—C21	119.4 (3)	O1—Cu—O2	88.33 (11)
C20—C21—H21A	109.5	N1—Cu—O2	96.41 (11)
C20—C21—H21B	109.5	N5—Cu—O2	71.28 (9)
H21A—C21—H21B	109.5	N4—Cu—O2	146.02 (9)
